# Wildfire as a major driver of recent permafrost thaw in boreal peatlands

**DOI:** 10.1038/s41467-018-05457-1

**Published:** 2018-08-02

**Authors:** Carolyn M. Gibson, Laura E. Chasmer, Dan K. Thompson, William L. Quinton, Mike D. Flannigan, David Olefeldt

**Affiliations:** 1 Department of Renewable Resources, University of Alberta, Edmonton, AB T6G 2R3 Canada; 2 Department of Geography, University of Lethbridge, Lethbridge, AB T1K 6T5 Canada; 3 Natural Resources Canada, Canadian Forest Service, Edmonton, AB T6H 3S5 Canada; 4 Cold Regions Research Centre, Wilfrid Laurier University, Waterloo, ON N2L 3C5 Canada

## Abstract

Permafrost vulnerability to climate change may be underestimated unless effects of wildfire are considered. Here we assess impacts of wildfire on soil thermal regime and rate of thermokarst bog expansion resulting from complete permafrost thaw in western Canadian permafrost peatlands. Effects of wildfire on permafrost peatlands last for 30 years and include a warmer and deeper active layer, and spatial expansion of continuously thawed soil layers (taliks). These impacts on the soil thermal regime are associated with a tripled rate of thermokarst bog expansion along permafrost edges. Our results suggest that wildfire is directly responsible for 2200 ± 1500 km^2^ (95% CI) of thermokarst bog development in the study region over the last 30 years, representing ~25% of all thermokarst bog expansion during this period. With increasing fire frequency under a warming climate, this study emphasizes the need to consider wildfires when projecting future circumpolar permafrost thaw.

## Abstract (web summary)

Future permafrost thaw may be underestimated unless effects of wildfire are considered. Here the authors show that wildfires in boreal permafrost peatlands influence soil temperature and seasonal thaw depth for several decades, and increase the rate of complete permafrost thaw along permafrost edges.

## Introduction

Permafrost thaw is expected to accelerate throughout the 21st century in response to a warming climate^1,2^. The rapid ecological and hydrological changes associated with permafrost thaw^3–5^ not only affect community infrastructure^6^, traditional land use, and water resources^7,8^, but are also expected to cause soil greenhouse gas emissions that potentially constitute a globally significant positive climate feedback^9,10^. Projections of future permafrost thaw may however be low as they do not consider the potential destabilization of permafrost following wildfire. Wildfire is the most widespread ecosystem disturbance in the boreal biome, with on average 25,000 km^2^ burned per year in Canada^11^. Forested peatlands are the dominant land cover in many boreal regions, and burn at least as frequently as other boreal forested ecosystems^12^. Peat stratigraphy in boreal peatlands show that past wildfires sometimes cause complete permafrost thaw that leads to land surface collapse and inundation, i.e. thermokarst^13^. However, an understanding is lacking of how much and for how long wildfires influence the soil thermal regime of permafrost peatlands, and of the spatial extents of thermokarst that develop due to wildfires. Understanding the role of wildfire as a driver of permafrost thaw is critical given recent trends of increased fire activity^14^.

**Fig. 1:**
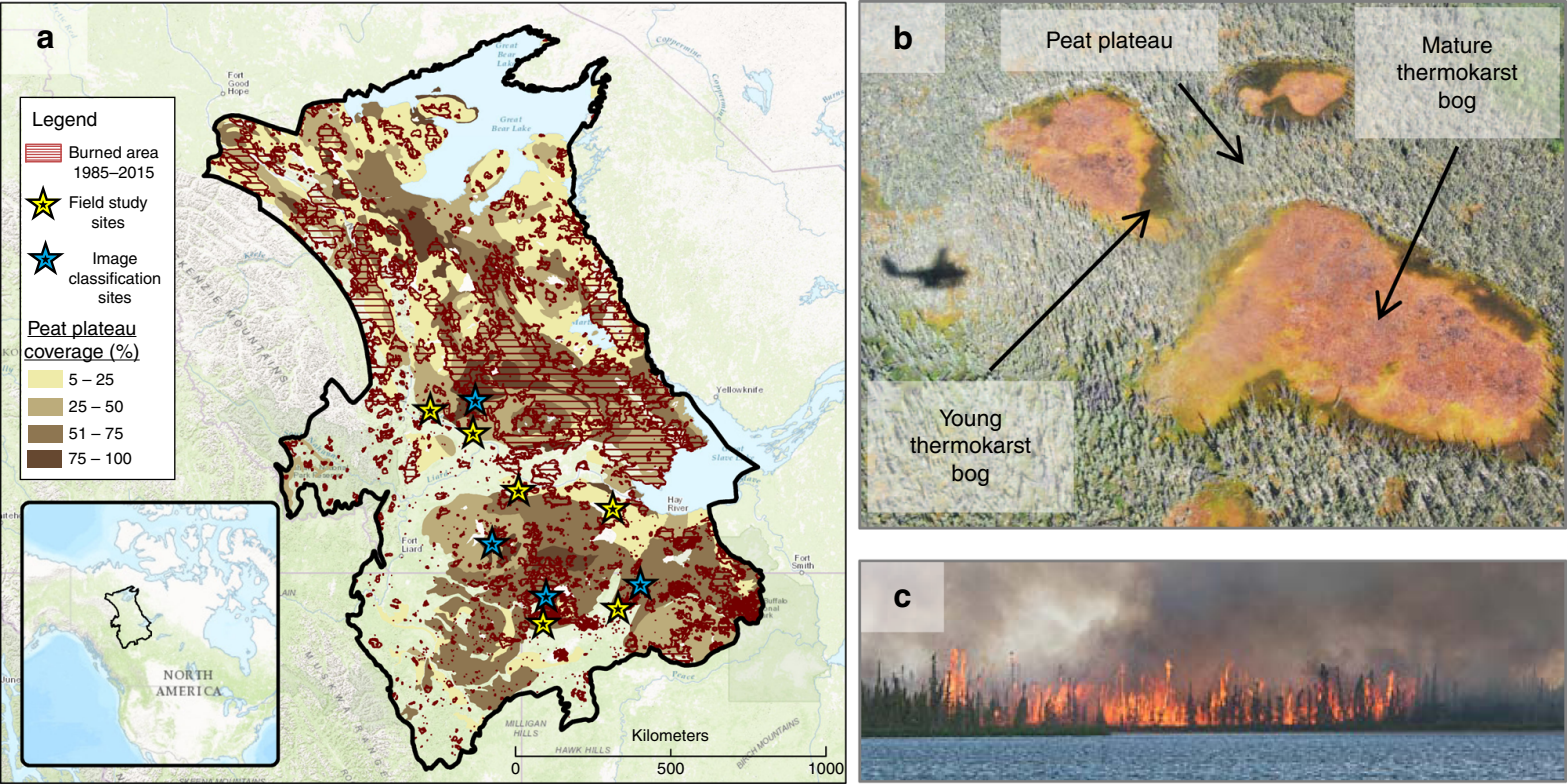
Wildfire in western Canadian peatlands. **a** Outline of the study region, defined as the discontinuous and sporadic permafrost zones^56^ within the Taiga Plains ecozone^59^. Shading shows coverage of peat plateaus as indicated by the distribution of histel soils^58^. Historical burned areas from the last 30 years are shown as hashed areas^57^. Yellow stars indicate locations where soil thermal regime was monitored, with one unburned and at least one burned site at each star location. Blue stars indicate locations where satellite image classification of young thermokarst bog extents were assessed within large peatlands partially affected by wildfire. **b** Example of a peatland characteristic for the study region, with three non-treed thermokarst bogs without permafrost surrounded by treed permafrost peat plateau. Distinct young thermokarst bog stages are visible as green areas along some peat plateau edges, resulting from recent permafrost thaw. Photo copyright Mason Stothart. **c** Example of a characteristic crown fire burning a peat plateau, near Fort Simpson, NWT, in 2014. Photo copyright Franco Alo

This study focuses on the sporadic and discontinuous permafrost zones within the Taiga Plains ecozone in western Canada, a ~400,000 km^2^ region that is representative of boreal regions with widespread peatlands (Fig. 1a). Peatlands cover nearly 40% of the study region, and peat accumulation in this area initiated following deglaciation ~9000 years ago^15^. Peat depths are today between 2 and 6 m, which constitutes some of the highest concentrations of soil organic carbon globally^16^. Permafrost started aggrading during the climate cooling after the Holocene thermal maximum ~5000 years ago, but became more widespread following further cooling 1200 years ago^17^. With current mean annual air temperatures between 0 °C and −5 °C, permafrost in this region is largely confined to peatlands^18^ due to the insulating properties of peat^19^. The relatively fragmented, thin, and warm permafrost in the region suggests that even minor perturbations to the soil thermal regime could cause extensive and rapid permafrost thaw.

Peatlands in the study region are dominated by peat plateaus with permafrost and thermokarst bogs without permafrost (Fig. 1b). Virtually all permafrost-affected organic soils, histels, in this region are found in peat plateaus^18^. Peat plateaus are elevated 1–3 m above the surrounding landscapes due to excess ground ice, which causes generally dry surface conditions and vegetation dominated by black spruce (*Picea mariana*) and Labrador tea shrubs (*Rhododendron groenlandicum*). The ground cover is dominated by lichens (*Cladina* spp.), and a <20% *Sphagnum* spp. cover. Black spruce forests burn preferentially in boreal western Canada^12^, and ~25% of peat plateaus in the study region have burned in the last 30 years (Fig. 1a, c, and Supplementary Fig. 1). Thermokarst bogs develop when peat plateaus undergo complete permafrost thaw, which often initiates as small peat plateau depressions which then expand as circular features and coalesce (Fig. 1b, and Supplementary Fig. 2). Thermokarst bog development causes the surface of peat plateaus to collapse, which initially leads to a young thermokarst bog stage with saturated soils and vegetation dominated by *Sphagnum riparium* and Rannoch-rush (*Scheuchzeria palustris*). As thermokarst bogs mature they accumulate new peat and thus become slightly drier, and vegetation becomes dominated by *Sphagnum fuscum* and ericaceous shrubs (Fig. 1b, and Supplementary Fig. 2). Previous studies have used repeat aerial photography, satellite image analysis, and tree ring analysis to show that the rate of thermokarst bog development has increased over the last few decades due to warming^20–23^. However, no analysis has been done to compare rates of thermokarst bog expansion within and outside historical fire scars.

**Fig. 2:**
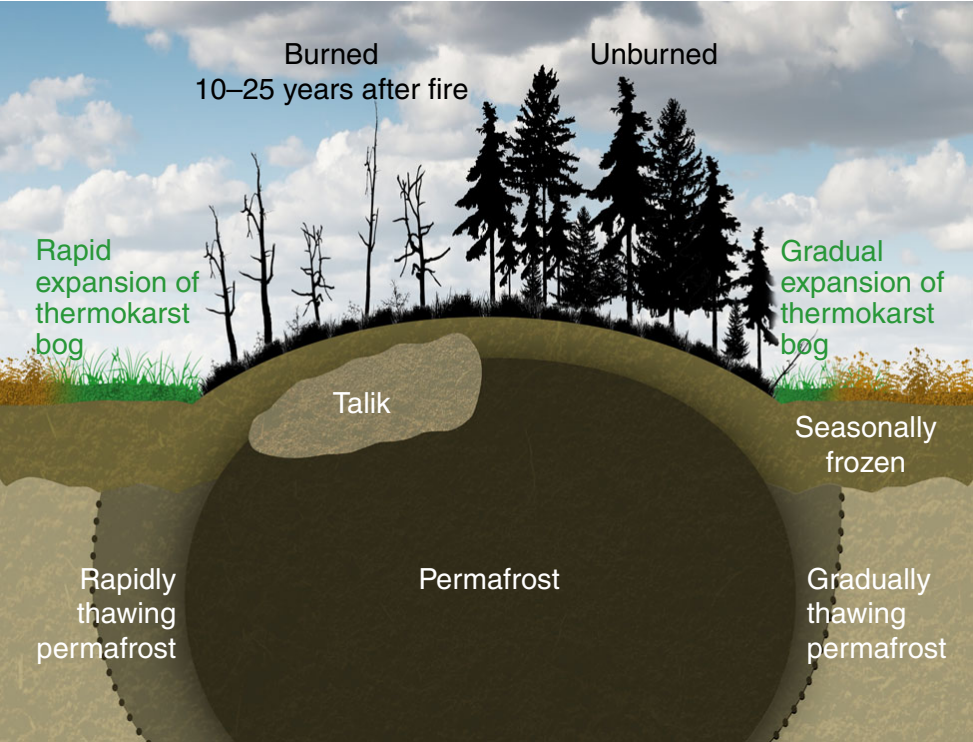
Simplified illustration of soil thermal states for peat profiles along transects from thermokarst bogs to peat plateaus, within and outside historical burn areas. Taliks are continuously thawed soil layers between the permafrost and the seasonally frozen layers

The objective of this study was to assess impacts of wildfire on permafrost stability and thermokarst bog development in boreal peatlands. Wildfire has been shown to cause deeper active layers^24–26^ and increased soil temperatures^25,27–29^ in Alaskan peatlands, but this has largely been attributed to the near complete combustion of relatively shallow peat profiles and greater thermal conductivity of the underlying mineral soils^26^. Potential impacts from wildfire on soil thermal regimes in our study region are thus required to act through other mechanisms given the greater peat depths. This may also affect both the severity of impacts as well as their temporal persistence. Given this, we hypothesized that impacts of wildfire on the soil thermal regime of peat plateaus are linked to vegetation controls on the surface energy balance, and thus predicted to observe warmer and deeper active layers that last for several decades after wildfire until vegetation recover. Furthermore, we hypothesized that a perturbed soil thermal regime on the top of peat plateaus increases the vulnerability to complete thaw at peat plateau edges, and thus predicted accelerated rates of thermokarst bog expansion within historical fire scars compared to unburned areas (Fig. 2). In order to test our hypotheses, we monitored soil thermal regimes at 16 peat plateau sites, ten of which had burned between 2 and 49 years prior to the study. We also assessed extents of recently developed young thermokarst bogs within and outside historical fire areas in four large peatlands that were partially affected by fire 20–30 years ago. Combining field measurements and remote sensing approaches allowed us to estimate an area of thermokarst bog development directly caused by wildfire over the last 30 years, and its relative contribution to overall thermokarst bog development in the study region.

## Results

### Effects of wildfire on peat plateau soil thermal regimes

The soil thermal regime at each of the 16 peat plateau sites (Fig. 1a, Supplementary Fig. 3, and Supplementary Table 1,) was characterized by monitoring air and soil temperatures at 40 cm depth for a full year, and via repeat measurements of depth to frost table at 100 point locations in 3 m spaced grids. Depths to frost table were measured up to seven times per site between May and late September. It was apparent from sudden large increases in depth to the frost table that many point locations had continuously thawed soil layers between the permafrost and the seasonally frozen layer, i.e. taliks (Fig. 2, and Supplementary Fig. 4a). We derived two measures of soil thermal regime from the within-site distribution of depth to the frost table in September; the proportion of point locations with taliks, and the active layer depth as defined by the typical depth to frost table at point locations without taliks (see Methods; Supplementary Fig. 4b, and Supplementary Tables 3, 4). Differences in soil thermal regimes were considered to be primarily due to differences in fire histories among sites, since sites all had similar peat depths, and current or pre-fire tree densities, and since the variability in mean annual air temperatures between −0.8 and −3.1 °C among unburned sites did not explain any of the variability in their active layer depth or talik coverage (*p* > 0.5, linear regressions) (Supplementary Table 1).

**Fig. 3:**
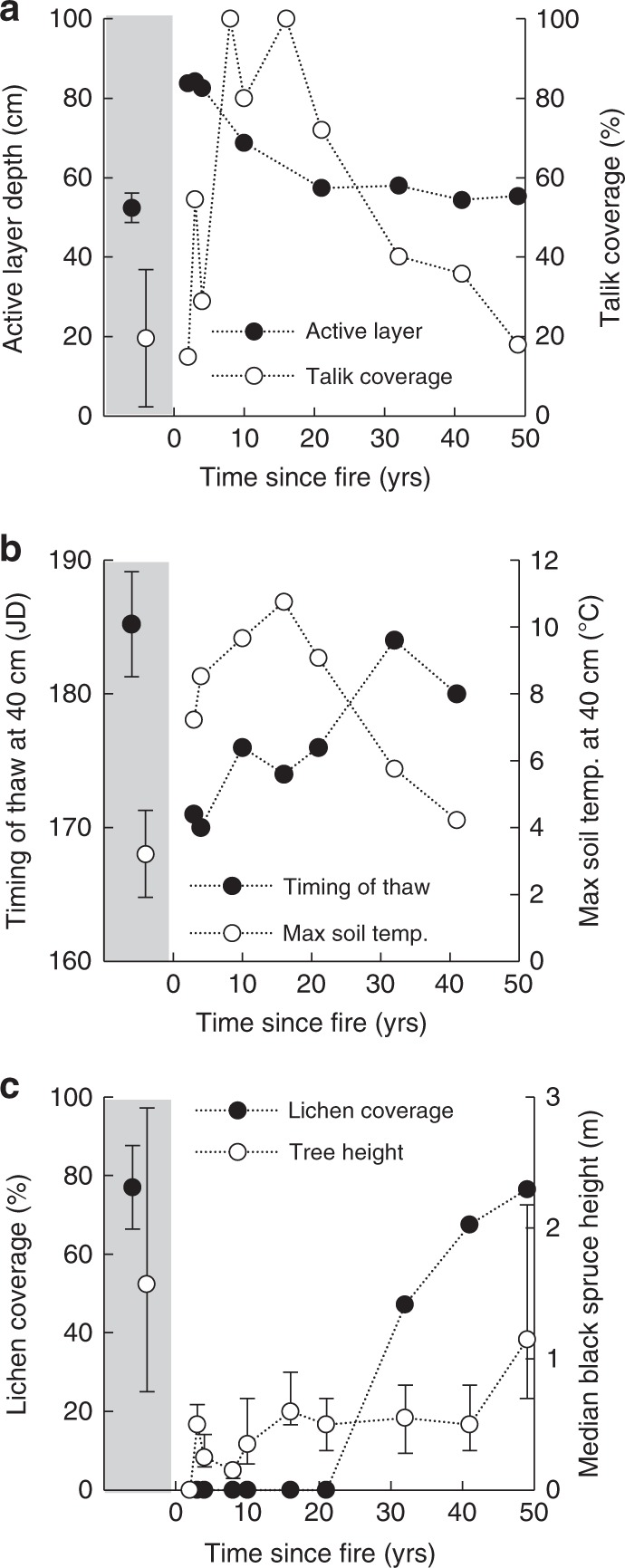
Trajectories of soil thermal regime and vegetation reestablishment on peat plateaus following wildfire. **a** Active layer depth (cm), and talik coverage (%). Taliks are continuously thawed soil layers between the permafrost and the seasonally frozen soil. No active layer estimates were possible for sites burned 7 and 16 years ago due to complete talik coverage. **b** Timing of thaw at 40 cm depth (Julian Date, JD), as indicated by timing of the first exceedance of 0.5 °C soil temperature, and the maximum annual soil temperature (°C) at 40 cm. Soil temperature loggers malfunctions at sites burned 7 and 49 years ago, and is missing. **c** Trajectories of vegetation reestablishment, including ground cover of lichens (%), and median height of black spruce trees (m), where error bars indicate interquartile range. In each panel, the average of 6 unburned sites is shown on the left with gray background, where error bars indicate ±1 standard deviation

We observed a >60% increase of the active layer in recently burned sites, from ~50 cm in unburned sites to ~85 cm in sites that burned within the last five years prior to the study (Fig. 3a). Recently burned sites had a deeper depth to the frost table than unburned sites already in June, and this difference increased throughout the summer and into fall (Supplementary Fig. 5). Accordingly, thaw at 40 cm depth occurred more than 2 weeks earlier at the recently burned than unburned sites, as indicated by soil temperature records (Fig. 3b). This suggested that fire was associated with increased downward ground heat flux throughout both summer and fall, possibly accompanied by an earlier initiation of the seasonal thaw development. We consider it likely that increased ground heat flux resulted from increased net radiation at the surface following the loss of shading from black spruce trees, and from reduced ground albedo^30^ as light-colored lichens had been replaced with black char (Fig. 3c, Supplementary Figs. 1 and 3). The observed effects of wildfire on active layer depth and rate of thaw throughout the summer were most apparent at recently burned sites, lessened at sites that burned 10–20 years ago, and were not detectable at older burn sites (Fig. 3a, b).

Our results further indicate that wildfire caused substantial development of taliks and increased deep soil temperatures. However, these effects were delayed and most pronounced at sites that burned 10–20 years ago, and only started to recover once active layer depths had returned to pre-fire conditions (Fig. 3a, b). Talik coverage increased from ~20% in unburned sites to between 70 and 100% in sites burned 10–20 years ago (Fig. 3a), while maximum annual soil temperature at 40 cm increased from between 2 and 5 °C to between 9 and 11 °C (Fig. 3b). The delayed response suggests that these effects represent a cumulative effect of wildfire which carries over from year to year. We speculate that wildfire increases heat penetration during the summer but also reduces soil heat loss during winter, which together prevents complete re-freeze of the active layer. This in turn allows for a continued deepening of the frost table and further talik expansion in the following year. Winter heat losses after fire may be reduced due to the loss of an intercepting tree canopy which causes increased snow depths and thus increased insulation^31^. Our results, indicating strong effects of wildfire on soil thermal regimes in peat plateaus are contrasting to the minor effects of wildfire found in more southern non-permafrost bogs^32^. This could be due to greater ground-cover dominance in non-permafrost bogs of *Sphagnum fuscum* hummocks that remain light-colored following fire^32^, or due to differing effect of fire on near-surface soil moisture and consequently on soil thermal conductivity.

Fire severity did not appear to have any influence on the post-fire soil thermal regime. Fire severity is generally higher during droughts and for fires that occur later in the season^33^. However, neither the Canadian drought code, which is a rating of the average moisture content of deep organic layers, nor the Julian date of the fires explained any of the variability in soil thermal regime among the burned peat plateau sites (*p* > 0.5, multiple linear regressions with active layer depth or talik coverage as dependent variables, and years since fire, Julian date, and drought code as independent variables) (Supplementary Table 1). The lack of an influence of fire severity may be due to the observed near-complete tree mortality and loss of lichens at all recently burned sites in this study (Fig. 3c and Supplementary Fig. 3). Hence, tree mortality and lichen loss appear to occur regardless of fire severity, which suggests that fire severity does not moderate the immediate effects of fire on shading or albedo. Fire severity furthermore did not appear to influence vegetation recovery. Variability in fire severity is known to affect timing and trajectory of vegetation recovery in some Alaskan ecosystems with relatively shallow soils, since high severity fires have the potential to cause almost total loss of soil organic matter^34,35^. While there likely was substantial variability among sites with regards to depth of burn among our sites, the thick organic soils of the peat plateaus prevented complete peat combustion^36^, which likely explain the consistent trajectory of vegetation recovery across burned sites (Fig. 3c).

The recovery of the soil thermal regime to pre-fire conditions coincided with vegetation reestablishment (Fig. 3a–c). Slow, decadal, vegetation recovery was likely linked to the dry, cold, and nutrient poor conditions on burned peat plateaus. Median height of regenerating black spruce trees remained <0.5 m even 10 years after fire, and lichens were absent in all sites that burned <20 years ago. Lichen coverage had only returned to pre-fire coverage in the two sites that burned >35 years ago, and tree height was not fully recovered even 50 years after fire. Influence of vegetation on shading, albedo, and snow pack dynamics during reestablishment may thus all have contributed to the recovery of the soil thermal regime^30,37,38^. The last aspect of the soil thermal regime to recover was the contraction of taliks, which occurred ~30 years after fire. Since taliks are likely to prevent effective heat loss from the permafrost core during winter, we consequently did not expect increased rates of thermokarst bog development due to complete permafrost thaw along peat plateau edges to last beyond 30 years after fire (Fig. 2).

### Effects of wildfire on thermokarst bog development

**Fig. 4:**
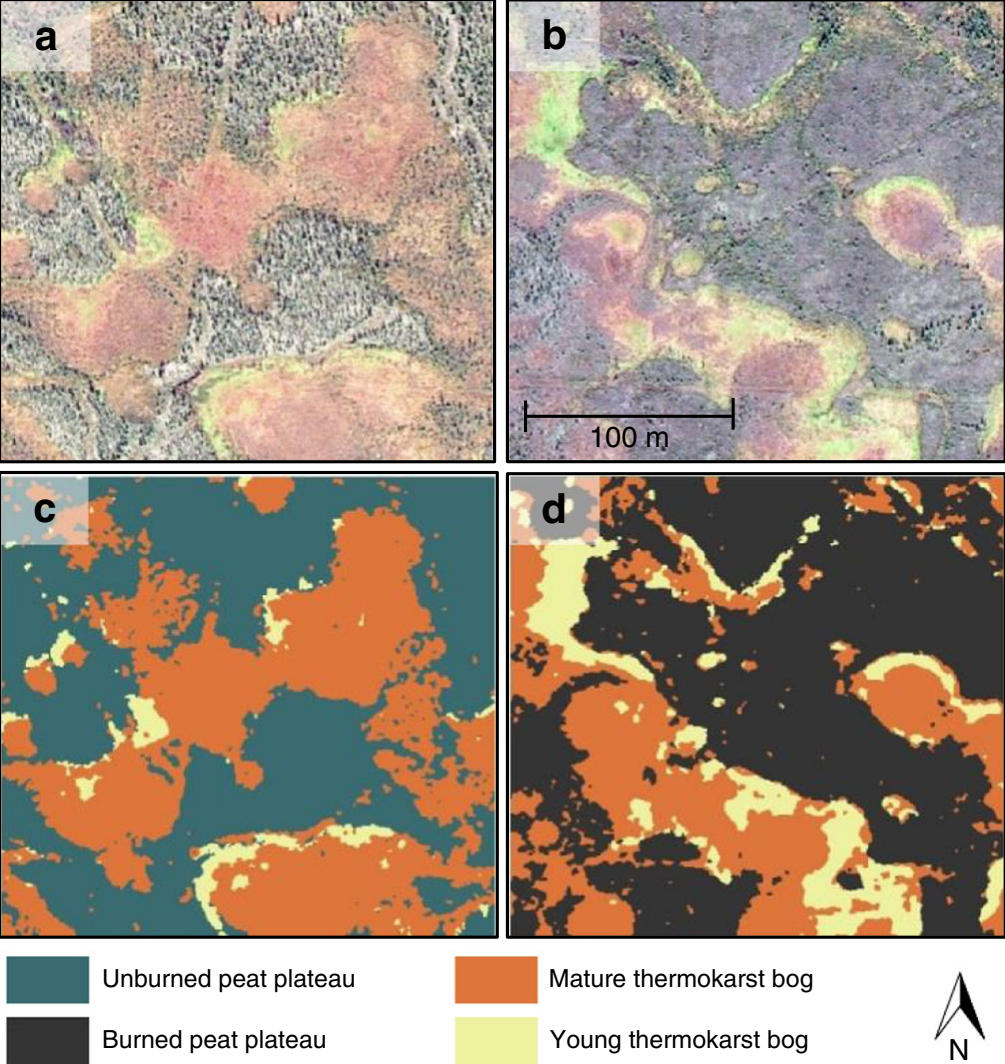
Classification of peat plateau, young thermokarst bog, and mature thermokarst bog using high resolution satellite imagery in peatlands partially affected by historical wildfires. **a** and **b** Examples of satellite imagery (WorldView-2, 0.6 m resolution) for 250 × 250 m sections within unburned and burned peatland parts, respectively. Satellite images were acquired in 2011, the burn occurred in 1987. **c** and **d** Examples of supervised classification of the unburned and burned sections, respectively

Young thermokarst bogs are generally found along thawing peat plateau edges, and they have a distinct vegetation composition that is clearly discernable in high resolution satellite imagery (Figs. 1b and 4). The transition from young to mature thermokarst bog is defined by a shift in dominance from *Sphagnum riparium* and *Scheuchzeria palustris* to *Sphagnum fuscum* and ericaceous shrubs^17^. Radiocarbon dating of cores from the study region show that this transition from young to mature thermokarst bog occurs between 60 and 140 years after the initial permafrost thaw that led to the development of the young thermokarst bog (Supplementary Table 5). In order to determine the effect of wildfire on thermokarst bog development, we chose to study four large peatlands that were partially affected by wildfire 20–30 years ago. The four peatlands were chosen for this analysis since the 20–30 years since fire coincided with the duration over which wildfire was found to influence peat plateau soil thermal regime, and thus likely also the period over which it would influence the rate of thermokarst bog development. Hence, we expected the majority of the cumulative effect of wildfire on thermokarst bog development to be accounted for by choosing sites that burned 20–30 year ago. However, analysis using the chosen sites cannot rule out effects of wildfire on thermokarst bog expansion extending beyond this time frame, and as such our analysis is potentially conservative. By assessing the spatial extents of peat plateaus, young thermokarst bogs and mature thermokarst bogs within each peatland we were able to estimate rates of thermokarst bog development over the last 60–140 years, a period which includes but extends beyond the influence of the more recent wildfires (Fig. 1a, Supplementary Table 2).

Supervised classification was done for between 30 and 65 sections (250 × 250 m) within burned and unburned parts of each of the four peatland sites (Supplementary Fig. 6, Supplementary Table 2). Burned and unburned peatland parts at each site were located <10 km apart. Precision of the supervised classification was assessed by comparison with field-determined dGPS locations of transitions between peat plateau, young thermokarst bog, and mature thermokarst bogs at the Zama site (Supplementary Fig. 7). The distance between field-determined ecological transitions and transitions in the supervised classification was within 1 m 80% of the time, and without bias, in both burned and unburned sections (Supplementary Fig. 8). The field validation thus showed that the supervised classification of young thermokarst bogs would be able to provide a both precise and unbiased measure of differences in thermokarst bog expansion between burned and unburned peatland parts.

**Fig. 5:**
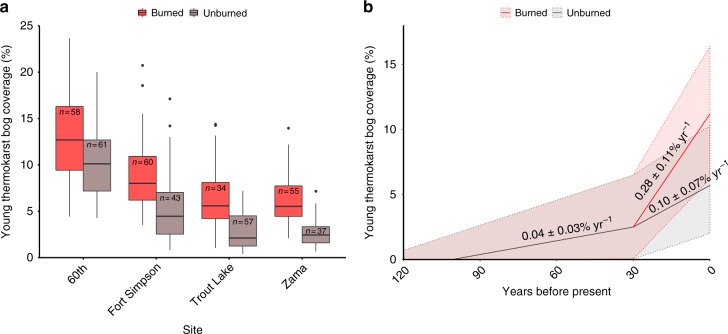
Effect of wildfire on permafrost thaw through development of young thermokarst bogs in western Canadian peatlands. **a** Current-day coverage of young thermokarst bogs in burned and unburned sections within four peatlands partially affected by wildfire 20–30 years ago. Box plots indicate median, interquartile range, and minimum and maximum young thermokarst bog coverage among classified 250 × 250 m sections, with number of classified sections indicated (*n*). A pairwise *t*-test showed a significant (*p* < 0.01) effect of fire on young thermokarst bog coverage. Sites are ordered left to right by likely decreasing mean annual air temperature, see text for justification. **b** Estimated historical development currently present young thermokarst bogs within peatlands in the study region. Young thermokarst bogs persist for 60–140 years before succession into mature thermokarst bogs^17^, suggesting that a significant proportion of young thermokarst bogs currently present developed prior to the more recent fires. Thermokarst bog development rates are stated with 95% CI (Methods), which are also indicated by shading

Average coverage of young thermokarst bogs within burned and unburned peatland parts was 8.6 and 5.3%, respectively, and the average difference between burned and unburned parts of paired sites was found to be 3.4 ± 0.5% (±1 SD) (Fig. 5a). A pairwise *t*-test indicated a significant influence of fire on young thermokarst bog coverage (*t* = −10.889, *p* < 0.01) when comparing average young thermokarst bog coverage in burned and unburned parts of the four paired sites. While the effect of wildfire was largely consistent between sites, we did observed a large variability in average young thermokarst bog coverage between unburned sites, ranging from 2.7 to 10.5% (Fig. 5a). We note that the lowest young thermokarst bog coverage was found at the higher elevation sites, Zama and Trout Lake (Supplementary Table 2). These sites are located at elevations ~300 m higher than the two other sites, and are indicated to have low subarctic climate in contrast to the lower elevation sites that are located in high boreal or mid-boreal ecoregions^18^. Field data confirmed that the Zama site had a colder climate than the Fort Simpson site during the year of our study, at −0.8 and −3.1 °C. While no direct climate data for the 60th Parallel site was available, this site was located both at a low elevation and in the southernmost part of the study region, and would thus be expected to have the warmest climate, thus possibly explaining the greatest young thermokarst bog coverage within the unburned areas among our four sites. This implied effect of climate on thermokarst bog development in unburned peatland parts^23^ contrasted with the lack of an observed difference in soil thermal regimes among unburned sites (Fig. 3). The greater young thermokarst bog coverage in burned than unburned parts at the Zama and Trout sites (+100–150% greater coverage in burned than unburned parts) than at the 60th Parallel and Fort Simpson sites (+30–70% greater coverage in burned than unburned parts) thus suggests that wildfire has had a relatively more pronounced influence on thermokarst bog expansion at colder sites (Fig. 5a).

The effect of wildfire on thermokarst bog development must however be greater than indicated by differences in current coverage of young thermokarst bogs, since much of the currently present young thermokarst bogs developed prior to the fires that occurred 20–30 years ago (Fig. 5b). In order to estimate rates of young thermokarst bog development at each of the four sites after the fire, expressed as percent of total peatland area developed into young thermokarst bogs each year, we made three assumptions. The first assumption was that burned and unburned peatland parts had similar rates of young thermokarst bog development prior to the fire. The second assumption was that young thermokarst bogs persist in the landscape 100 ± 50 years (95% confidence interval) before developing into mature thermokarst bogs (Supplementary Table 5), signifying that no young thermokarst bog currently present developed >150 years ago. The third assumption was that the rate of thermokarst bog expansion is likely to have increased over the last 30 years also in unburned peatland parts, due to ongoing climate change^20–22^ (see Methods for further details). Using these three assumptions, we estimated the rate of young thermokarst bog development prior to the fire, following fire in burned parts, and following fire in unburned parts for each of our four sites (Supplementary Fig. 9). Across our four sites, this analysis indicated that the rate of young thermokarst bog development within peatlands nearly tripled after fire, to 0.28 ± 0.11% yr^−1^ from 0.10 ± 0.07% yr^−1^ (95% CI) (Fig. 5b).

Rate of young thermokarst bog development can also be expressed as rates of peat plateau loss, i.e. as a change in young thermokarst bog area over time divided by the sum of peat plateau and young thermokarst bog land coverage. Across the four classified peatland sites, coverage of peat plateaus varied between 50 and 75% (Supplementary Table 2). As such, our estimated rates of peat plateau loss following fire were 0.39 ± 0.18% yr^−1^ and 0.16 ± 0.12% yr^−1^ (95% CI) in burned and unburned peatlands, respectively. Our resulting estimated rate of peat plateau loss within unburned peatlands was similar, but in the low range, to what other studies have estimated in the study region using historical image change detection (0.26–0.34% yr^−1^ peat plateau loss)^20,21^.

In order to estimate the total area of peat plateau loss due to thermokarst bog development within the study region over the last 30 years, we combined the distribution of peat plateaus, the distribution and timing of fires (Fig. 1), and our estimated rates and uncertainties of peat plateau loss within and outside burned areas (Methods). We assumed that the soils maps and fire maps have negligible errors, and that the effect of fire on the rate of thermokarst bog development lasts 30 years. Our remote sensing analysis cannot rule out any effects beyond 30 years after fire, and as such this is potentially a conservative measure of the effect of wildfire on thermokarst bog development. We estimated the total peat plateau loss to 9800 ± 4100 km^2^ (95% CI), of which 2200 ± 1500 km^2^, or ~23%, was directly attributed to the increased rate of thermokarst bog development that occurs following wildfire. This major role of wildfire occurred with wildfire having affected 25% of peat plateaus in the study region during the last 30 years. It is not clear from this study whether the role of wildfire as a driver of thermokarst bog expansion has become relatively more important, as it likely that thermokarst bog development due to both climate warming and increased fire occurrence have increased over the last 30 years compared to earlier periods. Overall, this study shows that wildfire has been a major driver of peat plateau loss due to accelerated thermokarst bog development in boreal western Canada, and thus likely a major cause of permafrost thaw in this region where a majority of the permafrost is found in peatlands.

## Discussion

In this study, we showed that wildfire in boreal peatlands within the discontinuous permafrost zone cause permafrost thaw through active layer deepening and talik expansion on peat plateaus, but also through accelerated thermokarst bog development along peat plateau edges. Our findings expand on earlier research that has highlighted the importance of wildfire as a driver of permafrost thaw^25,26,39,40^, particularly by being able to estimate the temporal horizon for effects of wildfire on soil thermal regimes, and being able to estimate the areal extents of complete permafrost thaw over the last 30 years caused by accelerated thermokarst bog development after wildfire. Permafrost thaw within peat plateaus (i.e. active layer deepening) appeared reversible under the recent climate since pre-fire soil thermal and vegetation conditions were able to recover fully after 30 years to pre-fire conditions. However, permafrost thaw at the edges of peat plateaus (i.e. thermokarst bog development) is considered irreversible given that these ecosystems undergo a complete successional shift to a new vegetation community that also includes strongly altered soil thermal and hydrological regimes. Thermokarst bog development has during the past few thousand years been cyclical in the study region at time-scales of centuries to millennia, with eventual permafrost aggradation in mature thermokarst bogs^13^. However, future permafrost aggradation in thermokarst bogs in this discontinuous permafrost zone study region is unlikely to be widespread since temperatures at high latitudes are increasing at twice the global rate^41^.

Projected impacts of permafrost thaw and thermokarst bog expansion in peatlands of western boreal Canada include increased stream flow^42^, increasing levels of dissolved organic matter^43^ and methylated mercury^44^ in surface waters, loss of habitat for the threatened woodland caribou, deteriorating access for traditional land-use^45^, and altered soil greenhouse gases^46–48^. The impact of permafrost thaw on soil carbon cycling and greenhouse gas exchange is one of the potentially most important biogeochemical feedbacks to anthropogenic climate change^49^, and both the altered soil thermal regime of peat plateaus and the increased rate of thermokarst bog expansion may be of importance^50,51^. Deeper active layers and warmer soils on peat plateaus for decades following wildfire may increase the soil carbon dioxide respiration rate, given the predominately aerobic soil environment. Conversely, increased methane emissions are caused by the land subsidence and anaerobic conditions that are associated with thermokarst bog expansion^52,53^. However, thermokarst bogs may have a long term cooling effect on the climate if the resulting anaerobic soil conditions along with *Sphagnum* moss productivity favor increased carbon accumulation^54^. Given the widespread occurrence of wildfire in the study region, projections of the future carbon balance of the study region will need to consider the effects of wildfire described in this study.

The results from this study are of relevance for other peatland-rich regions with permafrost. Peatlands with thermokarst landforms have been estimated to cover 1.4 × 10^6^ km^2^, or 8%, of the circumpolar permafrost region^55^, mainly in boreal but also in tundra regions. While the magnitude of effects from wildfire are likely to differ depending on the regional climate, peat plateaus under similar climates and fire regimes as described in this study are widespread also in boreal Alaska and in the Hudson Bay lowlands. While wildfire is a less important disturbance at the landscape scale in other regions under current climate, this might change under a future climate^56^. Hence, with recent and projected increases in wildfire activity due to climate change^14,57^, our results are thus of high relevance for our understanding the overall vulnerability of permafrost to thaw at the circumpolar scale, and its potential impacts.

## Methods

### Study region

The study region was defined by the discontinuous and sporadic permafrost zones within the Taiga Plains ecozone, a 431,000 km^2^ region. The Northern Circumpolar Soil Carbon Database^58^ was used to determine the distribution and coverage of permafrost peat plateaus within the study region, using the distribution of histel soils. Within the study area, practically all histels correspond to peat plateaus^59^. Each polygon in the Northern Circumpolar Soil Carbon Database has a specified coverage of histels between 0 and 100%. The area of peat plateaus affected by wildfire each year over the last 30 years was estimated by overlaying the histel distributions with burn area polygons from the Canadian National Fire Database^59^. Area of burned peat plateaus was estimated by summing up the product of percent histel coverage within each burn area and the size of each burn area.

### Peat plateau site selection and field data collection

Field data for this study were collected at six locations within the discontinuous permafrost zone of the Taiga Plains (Fig. 1a). Each location included one unburned peat plateau site and at least one historically burned peat plateau site, for a total of 16 sites (Supplementary Table 1). All peat plateau sites were identified using satellite imagery available in Google Earth in combination with burn area polygons from the Canadian National Fire Database, which includes information on the size and date of the fires that had affected the chosen burned sites (Supplementary Table 1)^59^. Locations were chosen to include burned sites that burned between 2 and 49 years prior to the study in 2016, but locations were also required to have nearby unburned sites and for all sites to be accessible either by foot from nearby roads or by short helicopter trips from Fort Simpson. While all locations were found in the southern part of the identified study region, each of the three level III ecoregions of within the study region (mid-boreal, high boreal, and low subarctic ecoregions) were represented by at least one study location, with the colder low subarctic climate ecoregion represented in southern part of the study region by a location at higher elevation (Zama).

Peat depth at each site was measured at the nearest peat plateau edge using a 3 m peat sampler (Eijkelkamp, the Netherlands) (Supplementary Table 1). Air and soil temperature at 40 cm was recorded hourly at each site for a year starting in September 2015. Two soil temperature loggers (HOBO 8k Pendant, Onset, US) were installed at 40 cm depth within each site under areas covered by lichen or char and away from any distinct hollows, in order to ensure consistent and comparable locations within all sites. Air temperature loggers were installed within a white temperature shield at ~1 m height.

Vegetation surveys were completed at each site by delineating a 50 m baseline transect along a randomly chosen geographic bearing. Three 30 m transects were established perpendicular to the 50 m transect: one at the center at the 25 m mark and two on either side of the center at randomly generated distances greater than 5 m away from the center point. Understory species abundance was estimated using the point intercept method^52^ along all three transects with a pin being dropped every meter. The number and type of species that touched the pin was recorded in each spot and species abundance was calculated using the following formula: Species abundance (% cover) = (number of hits per species/ total number of hits) × 100. Tree heights were visually estimated for all trees within 1 m of either side of each 30 m transect for a total sampling area of 180 m^2^.

Since a majority of burned sites were >10 years old, we could not directly assess fire severity consistently across burned sites, as indicated by depth of peat combustion. Instead we used seasonal timing of the fires and the applicable drought code for each fire as general indicators of fire severity. The seasonal timing of each fire was derived from the Canadian National Fire Database^59^, while the drought code data was obtained for each site from the nearest weather station for the day of the fire. Drought code data from the Fort Simpson weather station was obtained from the Canadian Forest Service, while the High Level and Zama weather station data was obtained from Alberta Agriculture and Forestry.

A 3 m spaced grid with 100 point locations was established at each site, away from nearby peat plateau edges, for determination of active layer thickness and talik coverage. For each point location we determined microtopographic position (hummock, hollow, or undefined microtopography), and dominant ground cover (lichen, *Sphagnum* spp., feathermoss, or char). Depth to frost table was measured using a 1.5 m soil probe at all sites in early June, mid-July, and early-September 2016. An additional four measurement occasions were carried out within this period at the Lutose location, which included an unburned site and sites burned in 2000, 2007, and 2012 (Supplementary Table 1). The higher frequency of depth to frost table measurements at these sites allowed for reliable direct detection of talik presence or absence, see below. Increased depth of frost table in microtopographic hollow positions on peat plateaus was found to be linked to subsurface drainage channels that occurred at spatial scales larger than the thaw depth grid^60^. Data on depth to frost table from hollows were thus excluded from further analysis, since their thaw depths were significantly greater than in other microtopographic positions (Kruskal–Wallis One-way ANOVA, *p* *<* 0.01). This excluded between 0 and 18 point locations among sites for further analysis (Supplementary Table 1).

Talik presence or absence at each point location in each site was determined using depth to frost table measurements done in late September. Point locations with greater than 100 cm depth to frost table were assumed to have taliks. This assumption was based on results from the four Lutose sites where higher frequency monitoring of depth to frost table allowed for reliable direct observation of talik presence or absence. At the four Lutose sites we determined talik presence if a point location had a >90 cm increase in depth to frost table between two measurement occasions (Supplementary Fig. 4a). A >90 cm increase in thaw depth in less than 3 weeks is not possible due to vertical heat conduction, and thus must result from the seasonal opening of a talik. We then compared these direct observations of talik presence or absence with the prediction of presence or absence based on the threshold of 100 cm depth to frost table in September. We found that the prediction had 92 and 97% accuracy for unburned and burned sites, respectively (Supplementary Tables 3, 4). With this validation, we applied the threshold of 100 cm depth to frost table in September to determine talik presence or absence across all sites.

Active layer depth at point locations without taliks was determined from the distribution of depth to frost table across point locations in September at each site. The distributions of depth to the frost table within each site generally had positive skewedness, partly due to presence of open taliks. We defined the active layer depth as the typical depth to the frost table, which was estimated using a kernel density estimator (Supplementary Fig. 4b). A kernel density estimator is a non-parametric method which establishes the probability density function of a random variable^61^. The depth with the greatest density in the probability function^62^ was thus used as our estimate of the typical depth to frost table at point locations without taliks (Supplementary Fig. 4b). The maximum density from the density function was deemed to give a better representation of the typical depth to frost table than either the arithmetic mean or mode (Supplementary Fig. 4b).

### Selection and land cover classification of large peatlands

Four large peatland complexes (>50 km^2^) were selected for land cover classification (Fig. 1a, Supplementary Table 2). Site selection was done by identifying large peatlands within the study region that were partially affected by wildfires between 1981 and 1996, and for which high resolution satellite images were available (WorldView-2, 0.6 m resolution). We obtained two 5 × 5 km images for each of the four peatland sites, one centered over a burned peatland area and one of an adjacent unburned area. Each pair of satellite images were taken on the same date in 2011, but specific dates varied among pairs between June and September. Geolocation of WorldView-2 images were checked using high resolution SPOT imagery. For our land cover classification, we first divided each WorldView-2 image into 500 sections (250 × 250 m) and visually excluded sections for further analysis if they contained clouds, shadows, roads, lakes, upland forests, or fens. Hence only sections exclusively with peat plateaus, young thermokarst bogs, and mature thermokarst bogs, were included in the supervised classification (Supplementary Fig. 6). The supervised classification was carried out for between 31 and 61 sections per image. Classification used three bands within the visible spectrum (blue 450–520 nm; green 520–600 nm; red 625–695 nm). Training sites for classification at each of the four sites were chosen based on our field knowledge of ecological transitions between peat plateau, young and mature thermokarst bogs (Fig. 4). Maximum likelihood classification was performed in ENVI based on the spectral signatures of these three classes. Post classification, a low pass filter was applied to reclassify individual or small numbers of pixels to the surrounding contiguous class, thereby achieving a smoother overall classification.

Assessment of the accuracy of the supervised classification was carried out in August 2016 at the Zama site, both within burned and unburned sections (Supplementary Table 2). The Zama site was the only of the four classified peatlands that was accessible by foot from roads. Using a differential GPS unit with 10 cm accuracy, we delineated transitions between peat plateau and young thermokarst bogs, and between young and mature thermokarst bogs. Point locations were collected approximately every 2 m along transitions within 8 thermokarst bogs (Supplementary Fig. 7). Accuracy of the supervised classification was assessed by the distance between the field-determined point locations and the nearest corresponding transition in the classified image (Supplementary Fig. 8).

### Estimating rates of thermokarst bog development

A pairwise *t*-test was used to test for differences in young thermokarst bog coverage between burned and unburned parts among the four sites. For the pairwise *t*-test, we used only the average young thermokarst bog coverage within classified 250 × 250 m plots (i.e. *n* = 4), thus avoiding issues of pseudoreplication when testing for a significant influence of wildfire on young thermokarst bog coverage. Using the spatial coverage of young thermokarst bogs at each of the four paired burned—unburned peatland sites, we then estimated three rates of thermokarst bog development; the rate of thermokarst bog development prior to the fire, the rate of thermokarst bog development in the unburned part of the peatland following the fire, and the rate of thermokarst bog development in the burned part of the peatland following fire. The central estimates of these thermokarst bog development rates, and their 95% CI, were based on three assumptions, visualized in Supplementary Fig. 9. First, we assumed that burned and unburned peatland parts had similar rates of young thermokarst bog development prior to the fire. Secondly, we assumed that young thermokarst bogs persist in the landscape 100 ± 50 years ( ± 95% CI) before developing into mature thermokarst bogs. This assumption is based on ^14^C and Pb dating of peat cores (Supplementary Table 5), and implies that all young thermokarst bogs currently present in peatland developed <150 years ago. Thirdly, we assumed that that the rate of thermokarst bog development likely have increased also in unburned peatland parts over the last 30 years, due to ongoing climate change^20–22^. For this third assumption we defined a 95% CI for the relative rate of thermokarst bog development in unburned peatland parts before and after the year of fire. The upper bound of this interval was defined by equal rates of thermokarst bog development before and after the year of fire, while the lower bound was defined as half the rate of the intervals’ upper bound. The third assumption thus implied roughly a doubled rate of thermokarst bog development in unburned peatlands over the last 30 years, which is consistent with observed increases in thermokarst bog development rates for unburned peatlands located in the study region^19^ (Supplementary Fig. 9b). Uncertainty estimates of the three thermokarst bog development rates were based on a boot-strap analysis with 5000 iterations. Each iteration used random values drawn from the normal distributions for the temporal persistence of young thermokarst bogs in the landscape and for the relative rate of thermokarst bog expansion rates before and after the fire in unburned peatland part—i.e., values based on assumptions 2 and 3 above.

### Scaling thermokarst bog development to study region

Thermokarst bog development between 1985 and 2015 within the study region was assessed using estimated rates of recent thermokarst bog development within and outside burn areas. The study region was estimated to contain 151,000 km^2^ peat plateaus, based on the distribution of histel soils^15^, of which an average of ~900 km^2^ was burned annually between 1955 and 2015^51^. For the scaling, we assumed that the soils maps and fire maps have negligible errors. We also assumed that the accelerated rate of thermokarst bog development lasted for 30 years after fire, i.e. that our analysis of peatlands that burned 20–30 years ago accurately captured the cumulative impact of wildfire on thermokarst bogs development. This meant that a peat plateau that burned in 1985 was assumed to have an accelerated rate of thermokarst bog development for the entire period between 1985 and 2015, while a peat plateau that burned in 2014 or in 1956 only had an accelerated rate of thermokarst bog development during one year of the period 1985–2015. In order to scale, we needed to express thermokarst bog development (new young thermokarst bog development expressed as a percent of total peatland area) as peat plateau loss (new young thermokarst bog development expressed as a percent of the total peat plateau and young thermokarst bog area within peatlands). Based on results from the four peatland sites where land cover classification was done, we estimated that the rate of peat plateau loss in areas that had not burned in the last 30 years was 0.16 ± 0.12% yr^−1^ (±95% CI) while loss rates of peatlands affected by wildfire was 0.23 ± 0.14% yr^−1^ greater (Fig. 5b). We applied the rate of peat plateau loss observed in unburned sections (0.16 ± 0.12% yr^−1^) to all peat plateaus in the study region, and the difference in rates of peat plateau loss between burned and unburned sections (0.23 ± 0.14% yr^−1^) to areas of peat plateaus affected by wildfire, taking into account the number of years within the last 30 years that this rate would apply. Uncertainties (95% CI) for peat plateau losses were estimated using a boot-strap analysis with 5000 iterations that used random values drawn from the normal distributions for rates of peat plateau losses in unburned areas and the difference in rate between burned and unburned areas. With this approach we were thus able yield estimates of both the total area of peat plateau loss due to thermokarst bog expansion, and the area of peat plateau loss that was a direct result of accelerated thermokarst bog expansion following wildfire.

### Data availability

The data that support the findings of this study are available from the corresponding author upon reasonable request.

## Electronic supplementary material

Supplementary Information

Peer Review File
