# Supplementary material for: Wildfire as a major driver of recent permafrost thaw in boreal peatlands
Source: Nat Commun. 2018 Aug 2;9:3041. doi: 10.1038/s41467-018-05457-1 (PMC6072743; doi:10.1038/s41467-018-05457-1)
Supplement: Supplementary file 1 — Supplementary Information [file 41467_2018_5457_MOESM1_ESM.pdf]

Supplementary Information:

Wildfire as a major driver of recent permafrost thaw in boreal peatlands.

Carolyn M. Gibson[1], Laura E. Chasmer[2], Dan K. Thompson[3], William L. Quinton[4], Mike D. Flannigan[1], and David Olefeldt[1*]

[1]Department of Renewable Resources, University of Alberta, Edmonton, AB, T6G 2R3, Canada

[2]Department of Geography, University of Lethbridge, Lethbridge, AB, T1K 6T5, Canada

[3]Natural Resources Canada, Canadian Forest Service, Edmonton, AB, T6H 3S5, Canada

[4]Cold Regions Research Centre, Wilfrid Laurier University, Waterloo, ON, N2L 3C5, Canada

[*]To whom correspondence should be addressed. E-mail: olefeldt@ualberta.ca.

Supplementary Table 1: Summary statistics for peat plateau sites where soil thermal regime was monitored. Date and size of fire derived from the Canadian National Fire Database. Number of point locations for measurements of depth to frost table is of out 100, after excluding point locations in microtopographical hollow positions (see Methods).

**Site**	**Site Coordinates**	**Elevation (m.a.s.l.)**	**Date of Fire**	**Drought Code[1]**	**Average Peat Depth (cm)**	**Thaw depth point locations (n)**	**Mean Annual Air Temp[2] (ºC)**
Samba Deh, 1967	61°21'18"N 121° 0'29"W	237	Jun 20, 1967	237	>300	100	-2.89
Camsell, 1975	62°17'59"N 122°38'8"W	293	Jun 24, 1975	264	230	92	-2.34
Zama, 1984	59°20'45"N 119°20'24"W	587	Jul 10, 1982	315	260	92	N/A[2]
Fort Simpson, 1995	61°59'45"N 121°23'45"W	186	Aug 18, 1995	696	>300	100	-0.15
Lutose, 2000	59°30'0"N 117°12'17"W	318	Jun 28, 2000	335	280	100	-0.58
Fort Simpson, 2006	61°59'24"N 121°23'51"W	185	Jul 22, 2006	338	200	100	-0.14
Lutose, 2007	59°25'2"N 117°17'4"W	324	Jun 13, 2007	287	>300	100	-0.91
Lutose, 2012	59°46'42"N 117° 3'40"W	299	Jul 9, 2012	536	>300	100	N/A[3]
Samba Deh, 2013	61°11'42"N 120° 5'25"W	290	Jun 27, 2013	225	290	99	-1.82
Kakisa, 2014	60°56'26"N 117°21'42"W	225	May 26, 2014	275	255	100	-1.91
Camsell, Unburned	62°14'46"N 122°34'23"W	262	-	-	230	99	-2.51
Samba Deh, Unburned	61°11'59"N 120° 6'50"W	287	-	-	290	98	-2.04
Lutose, Unburned	59°29'4"N 117°10'41"W	305	-	-	>300	95	-1.45
Zama, Unburned	59°22'9"N 119°19'16"W	584	-	-	260	100	-3.1
Kakisa, Unburned	61° 4'53"N 117°37'23"W	250	-	-	200	82	-1.57
Fort Simpson, Unburned	61°59'34"N 121°22'59"W	186	-	-	>300	96	-0.81

[1] Drought code a rating of the average moisture content of deep organic layers and can be used as a measure of fire severity.
[2]September 2015 – August 2016
[3]Temperature sensor malfunctioned

Supplementary Table 2. Summary statistics for peatlands sites where land cover classification was carried out. Results of classification are reported as average percent coverage of the three peatlands classes among classified sections, ± one standard deviation.

Site	Site Coordinates	Date of Fire (size, ha)	Elevation (m.a.s.l.)	Classified sections (n)	Peat Plateau coverage (%)	Young Thermokarst Bog coverage (%)	Mature Thermokarst Bog coverage (%)
Zama Unburned	59°20'N 119°19'W	Jul 10, 1984 (6,132)	584	55	74.3 ± 9.2	2.7 ± 1.3	23.0 ± 8.5
Zama Burned				37	66.8 ± 10.0	6.2 ± 2.8	26.7 ± 9.1
Trout Lake Unburned	60°26'N 120°51'W	Jun 28, 1996 (13,202)	559	34	70.3 ± 13.3	2.7 ± 1.9	23.9 ± 12
Trout Lake Burned				57	65.52 ± 8.4	6.3 ± 3.3	27.8 ± 8.2
Fort Simpson Unburned	62°25'N 121°20'W	Aug 3, 1995 (238,566)	260	60	49.4 ± 10.7	5.1 ± 3.4	44.7 ± 11.8
Fort Simpson Burned				43	52.9 ± 11.1	9.0 ± 3.9	37.7 ± 9.0
Sixtieth Unburned	59°51'N 116°33'W	Jul 12, 1981 (552,539)	314	58	55.0 ± 10.0	10.5 ± 3.9	34.3 ± 9.9
Sixtieth Burned				61	65.8 ± 8.4	13.0 ± 4.8	21.1 ± 8.0

Supplementary Table 3. Accuracy assessment for predictions of absence or presence of taliks within an unburned peat plateau site using September depths to frost table. Data is from the Lutose Unburned site (See Supplementary Table 1), where observed absence or presence of taliks was determined by repeated seasonal monitoring of thaw depths while predicted presence and absence was assessed using a threshold of >100 cm depth to frost table in September (See Supplementary Figure 4).

	**Predicted Absence of Talik**	**Predicted Presence of Talik**	**Sum**
**Observed Absence of Talik**	63	4	67
**Observed Presence of Talik**	4	24	28
**Sum**	67	28	95

Overall accuracy = 92%

Supplementary Table 4. Accuracy assessment for predictions of absence or presence of taliks within a burned peat plateau site using September depths to frost table. Data is from the Lutose 2012 Burned site (See Supplementary Table 1), where observed absence or presence of taliks was determined by repeated seasonal monitoring of thaw depths while predicted presence and absence was assessed using a threshold of >100 cm depth to frost table in September (See Supplementary Figure 4).

	**Predicted Absence of Talik**	**Predicted Presence of Talik**	**Sum**
**Observed Absence of Talik**	36	2	38
**Observed Presence of Talik**	1	55	56
**Sum**	37	57	95

Overall accuracy = 97%

Supplementary Table 5. Dating of transitions between plateau peat (sylvic) and young thermokarst bog (*Sphagnum spp*) peat in cores collected from current young and mature thermokarst bog sites. Note that these cores were not collected at the very transitions between young and mature thermokarst bogs, but rather centrally within young and mature thermokarst bogs, respectively, and thus in order to assess the persistence of young thermokarst bogs in the landscape, we are interested in seeing the maximum age of transitions within current young thermokarst bogs and the minimum age of transition in current mature thermokarst bogs. For ^{14}C dating, we report time since thaw as years before present (where the present is 1955) + 60 yrs.

Core type and ID	Location	Depth of transition (cm)	Time since thaw (yrs ago)	Transition dating method	Reference
Young thermokarst bog cores					
SC1-E	61°18'N 121°18'W	0	25	Aerial photo	Pelletier et al. 2017
SC1-YB	61°18'N 121°18'W	61	130	^{210}Pb	Pelletier et al. 2017
SC2-YB	61°18'N 121°18'W	60	125	^{210}Pb (Model)	Olefeldt et al. in prep
SC3-E	61°18'N 121°18'W	29	60	^{210}Pb (Model)	Olefeldt et al. in prep
SC3-YB	61°18'N 121°18'W	55	105	^{210}Pb (Model)	Olefeldt et al. in prep
SC4-E	61°18'N 121°18'W	47	145	^{210}Pb	Olefeldt et al. in prep
SC4-YB	61°18'N 121°18'W	62	120	^{210}Pb	Olefeldt et al. in prep
L1-IB	59°28'N 117°10'W	42	141[1]	^{14}C	Heffernan et al. in prep
Maximum time since thaw			*145*		
Mature thermokarst bog cores					
SC1-OB	61°18'N 121°18'W	130	595[1]	^{14}C	Pelletier et al. 2017
SC2-OB	61°18'N 121°18'W	89	432[1]	^{14}C	Olefeldt et al. in prep
SC3-OB	61°18'N 121°18'W	66	208[1]	^{14}C	Olefeldt et al. in prep
SC4-OB	61°18'N 121°18'W	120	652[1]	^{14}C	Olefeldt et al. in prep
L1-OB	59°28'N 117°10'W	89	427[1]	^{14}C	Heffernan et al. in prep
Minimum age of transition			*208*		

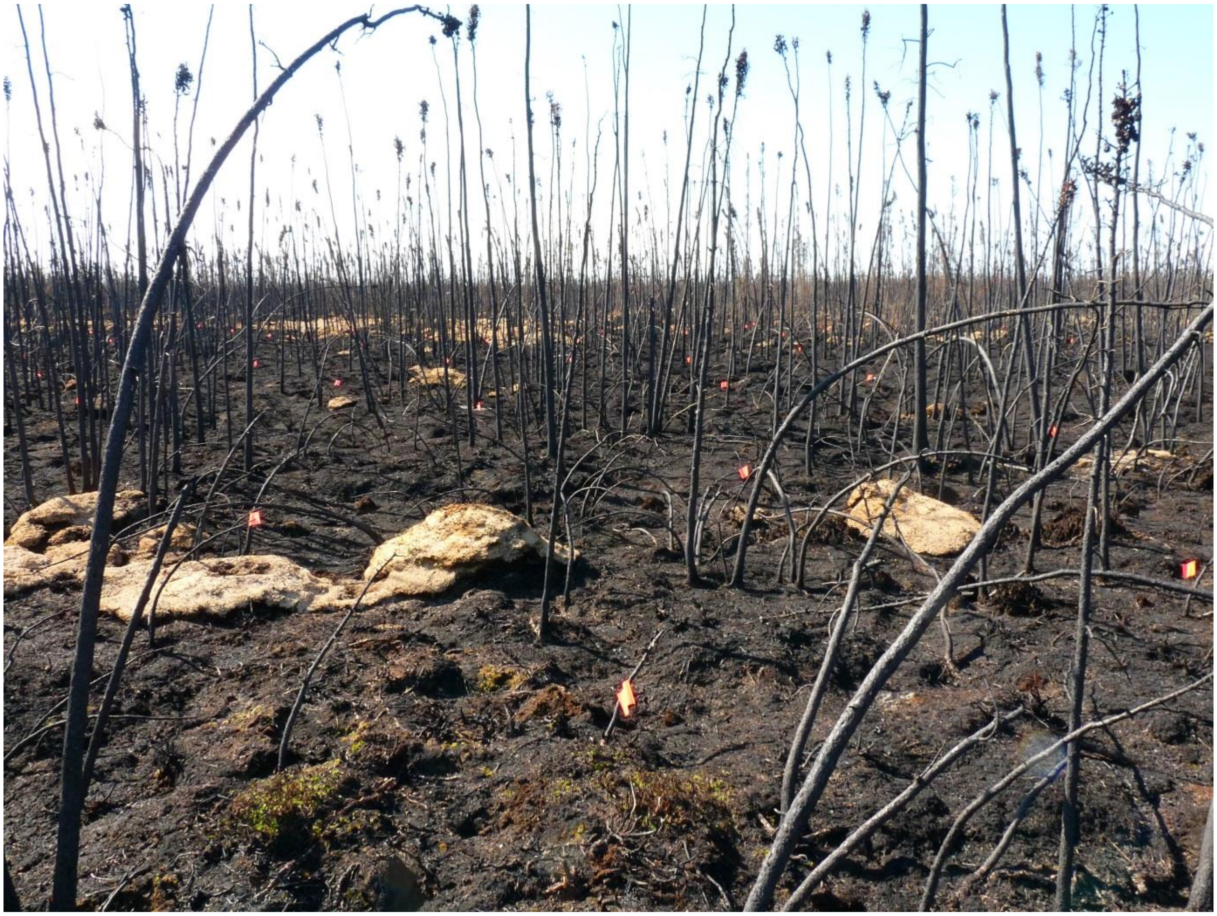

Supplementary Fig. 1. Soil thermal regime monitoring at the Kakisa burned peat plateau site (Supplementary Table 1). The site burned in 2014, two years prior to the study. Each peat plateau site had a 3 m spaced grid with 100 point locations marked with flags where depth to frost table was measured.

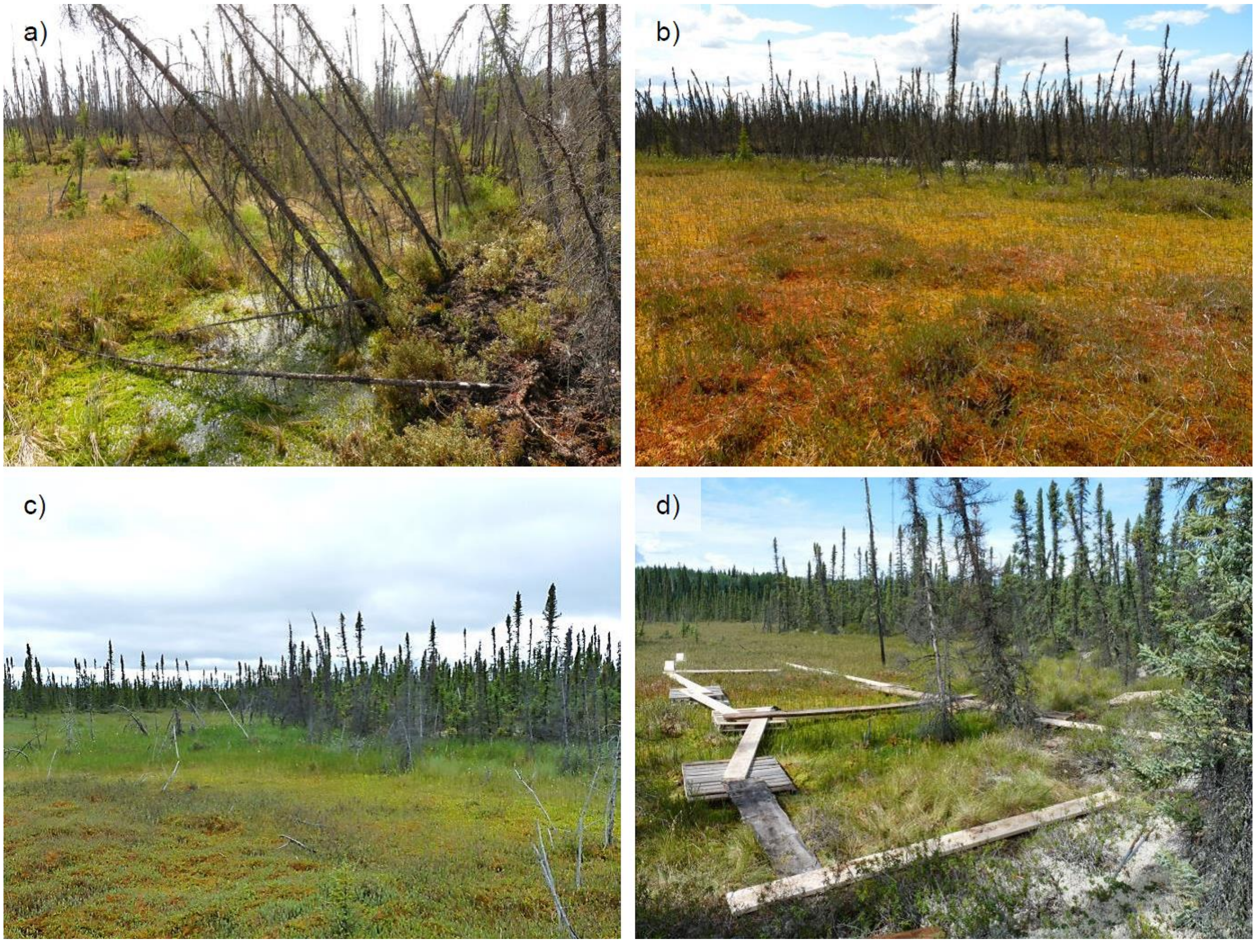

Supplementary Fig. 2. Example of transects from peat plateau, through young thermokarst bog, to mature thermokarst bogs. a) Lutose 2012 burned site, b) Samba Deh 2013 burned site, c) Fort Simpson unburned site, d) Lutose unburned site (See Supplementary Table 1).

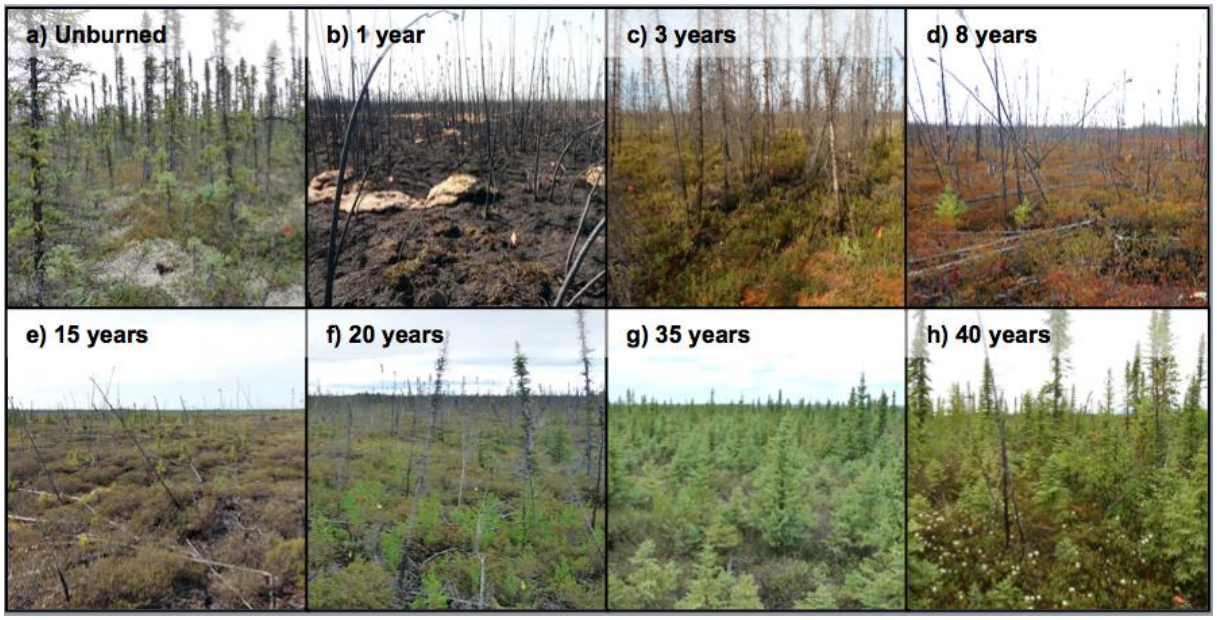

Supplementary Fig. 3. Vegetation reestablishment at select peat plateau sites used in this study to assess long term effects of fire on soil thermal regime.

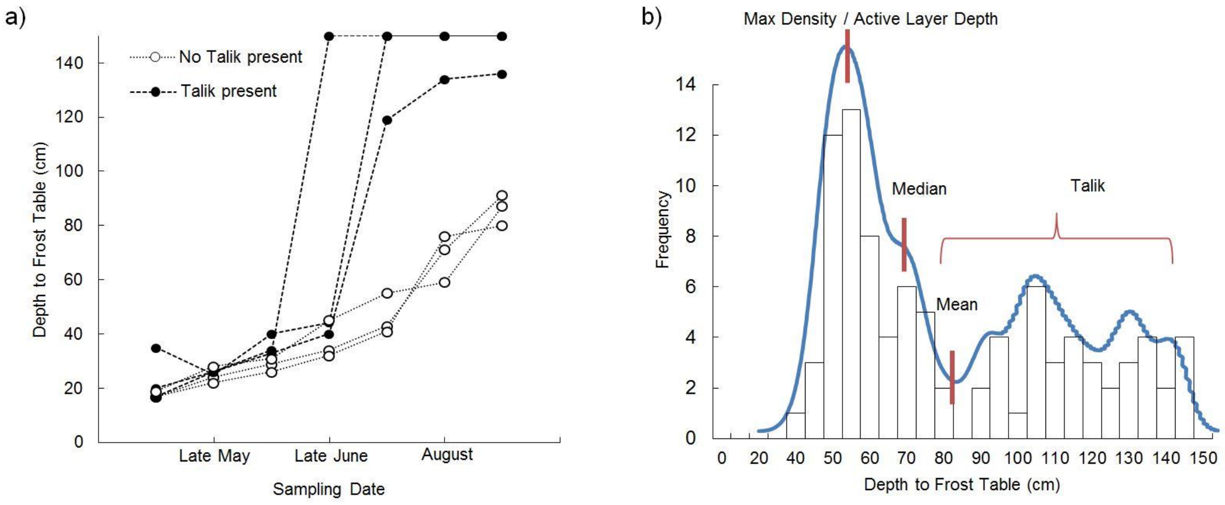

Supplementary Fig 4. Determination of talik presence or absence, and estimation of active layer depth for point locations where taliks were absent. A) Example of seasonal development of the thawed soil layer at 6 representative point locations at the Lutose 2012 burned site. The soil probe used was 150 cm long; hence measures of 150 cm indicate that no frost table was reached. We classified point locations as having taliks if there was a >90 cm increase in thaw depth between two monitoring occasions, as exemplified here by three point locations. B) Histogram of September point location depth to frost table measurements in 5 cm intervals from the Fort Simpson 1995 site, overlaid by a gaussian kernel density estimation. All point locations with September thaw depths >100 cm were assumed to have taliks, an assumption that was tested against observed talik presence (See Supplementary Tables 3 and 4). We used the maximum density of the density function as our estimate of the typical active layer depth for point locations where taliks were absent, which was deemed a better measure than either the mean or mode of the data.

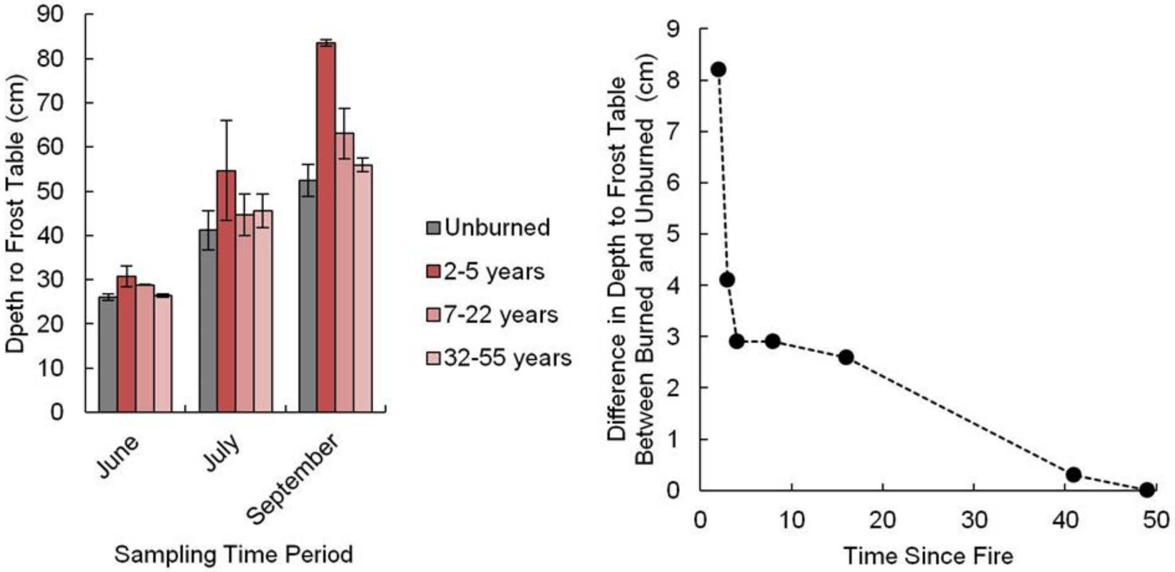

Supplementary Fig. 5. Differences in the seasonal development of the thawed soil layer between unburned and historically burned sites. A) Thaw depths for sites grouped by time since fire are shown ±1 standard deviation, with n = 3 for sites burned 2-5 years before the study, n = 4 (7-22 years), and n = 3 (32-55 years). Point locations where taliks later developed were included for June and July dates. B) Absolute difference in depth to frost table between burned and unburned sites in early June.

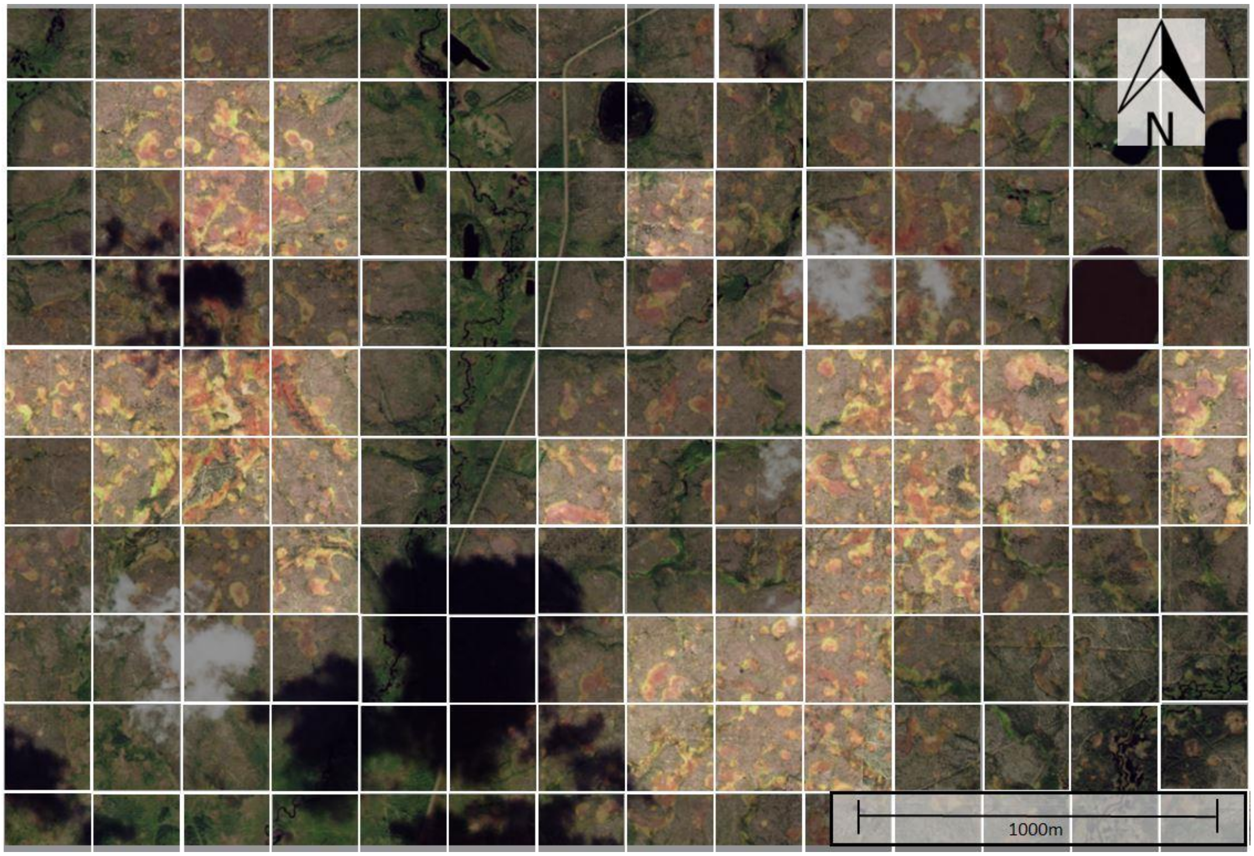

Supplementary Fig 6. Example of Worldview 2 satellite image used for peatland land cover classification. Supervised classification was carried out for 250 x 250 m sections of peatlands that were visually assessed to only contain peat plateau, young thermokarst bog, and mature thermokarst bogs. Sections containing fens, upland forests, ponds, clouds, or shadows were excluded from analysis and are shown in this figure with grey shading. This image is from the Zama site that burned in 1984, with the image acquired in 2011.

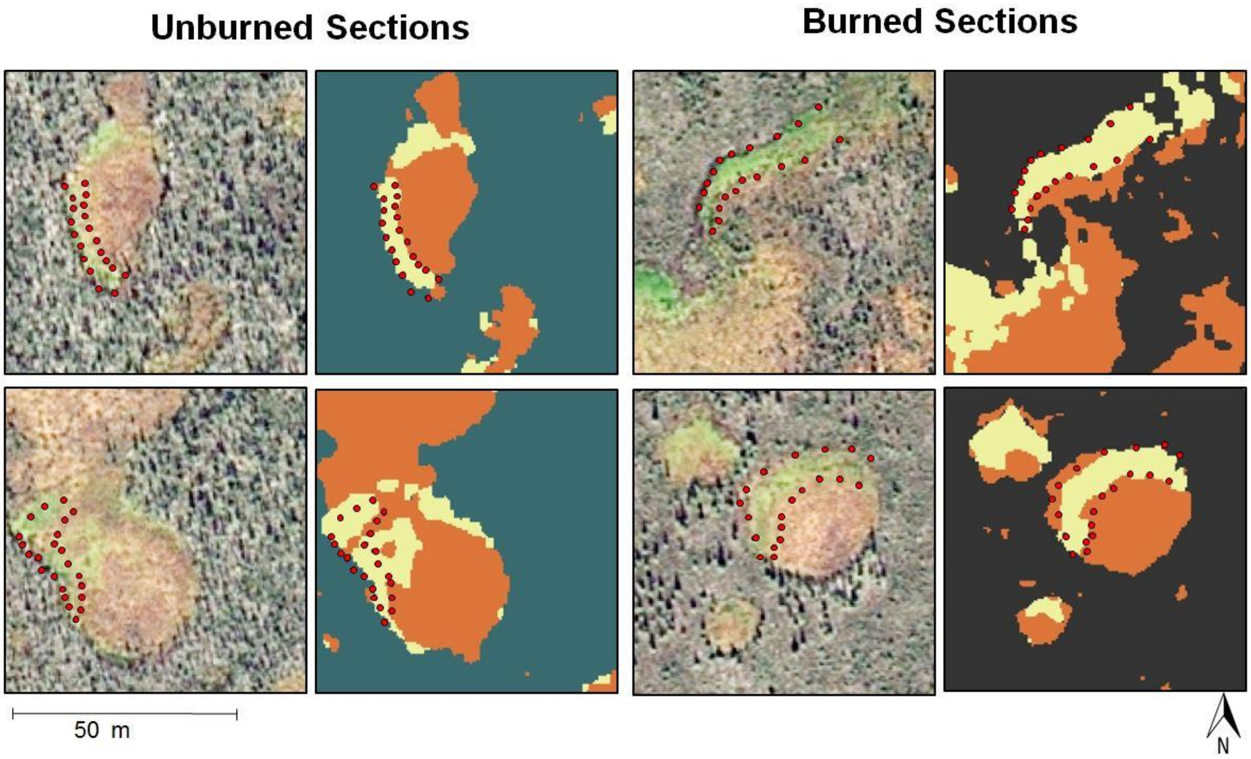

Supplementary Fig 7. Field validation of transitions between plateau and young thermokarst bog, and between young thermokarst bog and mature thermokarst bog as determined through the land cover classification. World View 2 Satellite imagery (75 × 75 m excerpts) are shown on left with corresponding classification to the right. Yellow areas represent areas of young thermokarst bogs, orange areas are mature thermokarst bogs and green/brown areas are unburned/burned peat plateaus, respectively. Overlain points were collected using a dGPS unit (10 cm horizontal accuracy) in the field.

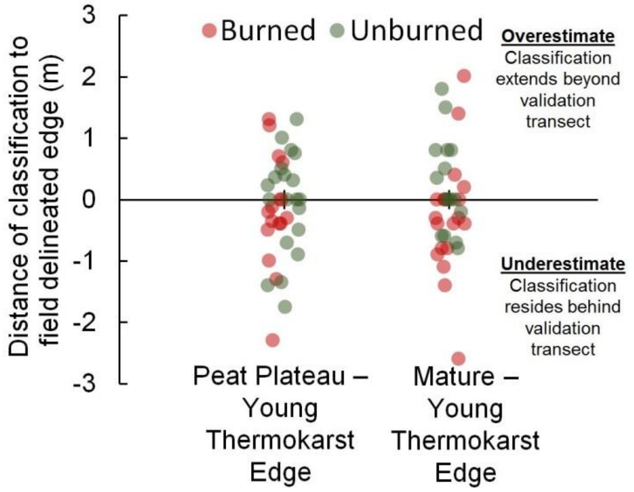

Supplementary Fig. 8. Assessment of the precision in the supervised classification of peatland land cover. Distances shown for differences between positions of classified and field determined transitions between peat plateau and young thermokarst bog, and between mature and young thermokarst bog. Positive values were defined for when the classification determined the transition to be located outside the field delineated young thermokarst bog area, while negative values correspond to when the classification determined the transition to be within the field delineated young thermokarst bog area.

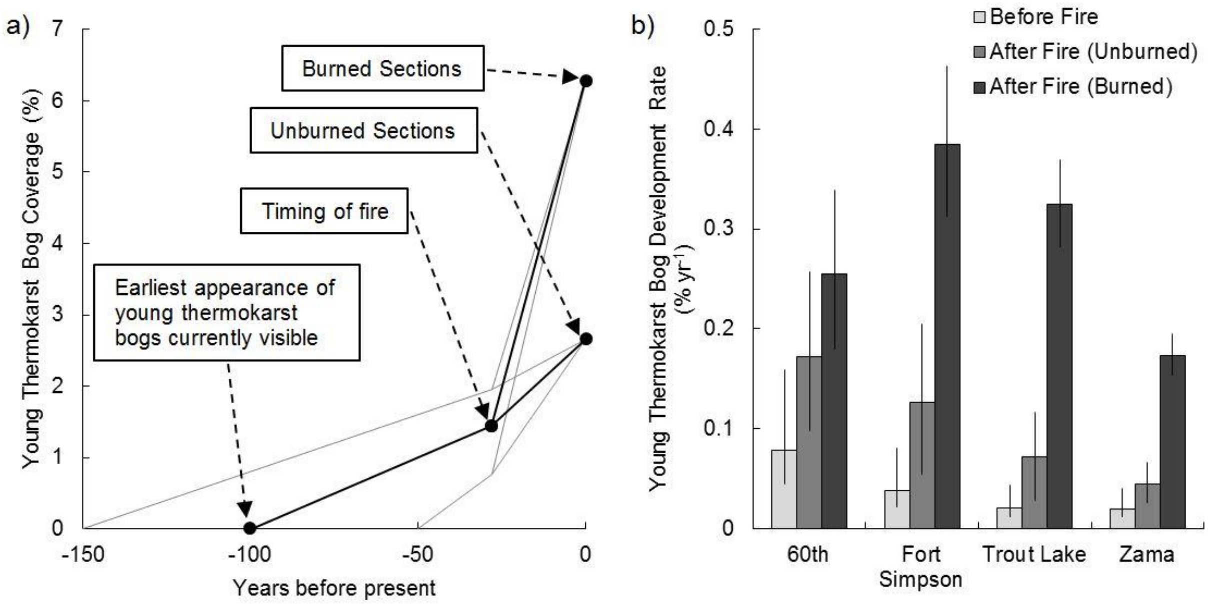

Supplementary Figure 9. Estimated developmental trajectories of young thermokarst bogs currently present at four peatland sites partially affected by wildfire 20 to 30 years ago. A) Trajectories of young thermokarst bog development, based on a set of assumptions: initiation of young thermokarst bog development 100 ± 50 years ago, similar development rates prior to the fire in sections later affected or not by the fire, and accelerated young thermokarst bog development in sections unaffected by fire due to climate change. Boot strap analysis yields the 95% confidence intervals of the development trajectories, indicated by grey lines. See Methods for further details. B) Estimates of young thermokarst bog development rates prior to fire, and after fire for burned and unburned sections, at each site with 95% confidence intervals based on the boot strap analysis.
